# Supplementary material for: Expanded HPV Genotyping by Single-Tube Nested-Multiplex PCR May Explain HPV-Related Disease Recurrence
Source: Microorganisms. 2024 Nov 15;12(11):2326. doi: 10.3390/microorganisms12112326 (PMC11596377; doi:10.3390/microorganisms12112326)
Supplement: Supplementary file 1 [file microorganisms-12-02326-s001.zip › microorganisms-3277391-supplementary.pdf]

microorganisms

Supplementary Materials: The following are available online at https://www.mdpi.com/article/10.3390/microorganisms12112326/s1,

Table S1: Comparative analysis of Nested Multiplex PCR (NMPCR), Cytopathology, and Hybrid Capture II (HC2) assays from genital swabs collected from 40 consecutively recruited women during routine HPV infection monitoring at a gynecology clinic in São Luís, MA, Northeast Brazil.

Case	NMPCR (HPV subtypes)	Risk Classification			Cytopathology	HC2
		LR	MR	HR		
**04**	*31,56,73,74*	-	1	3	*CIN I*	*HPV-B*
**05**	*06,34,42,43,55,73*	3	1	2	*Inflammation*	*HPV-A/-B*
**08**	*61,67*	-	1	1	*CIN III*	*HPV-B*
**09**	*42,44,70*	2	-	1	*CIN I*	*HPV-A/-B*
**20**	*06, 51*	1	-	1	*CIN I*	*HPV-A/-B*
**21**	*74*	-	1	-	*Inflammation*	*HPV-A/-B*
**23**	*39,52,71,81*	-	2	2	*Inflammation*	*HPV-A/-B*
**24**	*16,39,81*	-	1	2	*CIN I*	*HPV-A/-B*
**29**	*44,53,73,74,84*	1	2	2	*Inflammation*	*HPV-A*
**40**	*16*	-	-	1	*CIN III*	*HPV-B*
**06**	*N*	-	-	-	*Normal*	*N*
**11**	*N*	-	-	-	*Normal*	*N*
***16**	*N*	-	-	-	*CIN II*	*N*
***26**	*N*	-	-	-	*CIN I*	*N*
**30**	*N*	-	-	-	*Inflammation*	*N*
**33**	*N*	-	-	-	*Normal*	*N*
**36**	*N*	-	-	-	*Normal*	*N*
**39**	*N*	-	-	-	*Normal*	*N*
**01**	*61,73*	-	1	1	*Normal*	*N*
**02**	*34,39,44,51,52,53 54,55,66,67,73,74*	1	3	8	*CIN I*	*N*
**03**	*44,45,51,52,62,66 73*	1	1	5	*Inflammation*	*N*
**10**	*06, 30,53,62,81*	1	2	2	*CIN I*	*N*

**12**	39,52,53,66,67,72	-	1	5	Inflammation	N
**13**	16,34,39,45,53,62 66,73,74,81	-	3	7	Inflammation	N
**14**	16,45,52,66,67,74	-	1	5	CIN I	N
**15**	52,53,61	-	1	2	Inflammation	N
**17**	26,53,74	-	1	2	CIN I	N
**18**	42,44,73,84	2	1	1	CIN I	N
**19**	16,39,53,73	-	-	4	CIN III	N
**22**	16,42,51,73,74	1	1	3	Inflammation	N
**25**	69,83	-	1	1	CIN II	N
**27**	26,30,39,59,61,81 84	-	3	4	Normal	N
**28**	53,58,74	-	1	2	CIN I	N
**31**	44,74	1	1	-	Inflammation	N
**32**	06	1	-	-	Normal	N
**35**	06,30,31,42,61,66 73	2	1	4	Inflammation	N
**37**	16	-	-	1	Warts	N
**38**	72	-	1	-	Vulvar lesion	N
**07**	N	-	-	-	Inflammation	HPV-A/-B
**34**	N	-	-	-	Inflammation	HPV-A/-B

Highlighted in dark gray are the patients (N=10/40; 25%) who tested positive in both the NMPCR and HC2 assays.

No highlight indicates patients (N=8/40; 20%) who tested negative in both the NMPCR and HC2 assays.

Highlighted in medium gray are the patients (N=20/40; 50%) who tested positive with the NMPCR assay but negative with the HC2 assay.

Highlight in light gray are the patients (N=2/40; 5%) who tested negative with the NMPCR assay but positive with the HC2 assay.

LR: Low-Risk; MR: Medium-Risk; HR: High-Risk; N: Negative.

** indicates patients (2/40; 5%) who tested negative in both the NMPCR and HC2 assays, but were positive for cervical intraepithelial neoplasia lesions.*

CIN indicates Cervical Intraepithelial Neoplasia lesions (I, II and III).

Table S2. Reported prevalence of HPV DNA in female genital samples from epidemiological studies.

References	Detection methods	N	Population	HPV genotypes	Genital specimens (Prevalence %)
Present study (2024)	*Degenerated/ modified MY11/09 + NMPCR assay*	*5,263*	*Women who underwent routine gynecological examination (Brazil)**	*06 low-risk 11 medium 23 high-risk*	*Endocervical and ectocervical swabs (58.9%)*
Basilettti et al., 2022 [34]	*COBAS HPV systems*	*3,338*	*ESTAMPA** study (Women attending cervical screening)*	*-*	*Cervical samples (15%)*
Wendland et al., 2020 [31]	*Linear Array (Roche)*	*6,388*	*5,268 women/1,120 men randomly recruited (Brazil)*	*13 high-risk and 24 others HPV genotypes*	*Cervical samples collected using the Qiagen HC2 DNA collector (54.6%); scrotal surface, glans penis/coronal sulcus, and penile shaft samples collected using a Dacron swab (51.9%)*
Zhao et al., 2018 [35]	*DH3 Hybrid Capture and COBAS HPV systems*	*10,699*	*Women (China)*	*14 high-risk*	*Cervical scrapes (9.9%)*
Ramas et al., 2013 [36]	*MY09/11 PCR + RFLP*	*568*	*Women (Uruguay)*	*38 genotypes*	*Cervical exfoliated cells (42.0%)*
Amaro-Filho et al., 2013 [37]	*PGMY11/09 PCR + INNO-LiPA HPV Genotyping*	*130*	*87 women with invasive cervical cancer and 43 controls, Southeastern (RJ, Brazil)*	*07 low-risk 03 medium 18 high-risk*	*Cervical biopsy or hysterectomy (29.3% and 95.4%, respectively)*
Aruhuri et al., 2012 [38]	*GP5+/6+ PCR assay*	*998*	*Women (Vanuatu, South Pacific)*	*31 low-risk 13 high-risk*	*Cervical exfoliated cells collected using a Cervex-Brush (28.4%)*
Kondo et al., 2012 [39]	*PGMY-CHUV assay*	*326*	*Women with abnormal Pap smears (Japan)*	*05 low-risk 07 medium 19 high-risk*	*Cervical exfoliated cells collected using a Cervex-Brush (94.0%)*
Martin et al., 2011 [40]	*PGMY09/11 PCR + Dot blot hybridization*	*2,461*	*Women (Spain)*	*05 low-risk, 02 medium 14 high-risk*	*Cervical exfoliated cells collected using a cytobrush (43.2%)*
Fernandes et al., 2009 [32]	*MY11/09 PCR + Dot blot hybridization*	*250*	*Women (Northeastern, RN, Brazil)*	*07 low-risk 12 high-risk*	*Exfoliated cells collected using a cytobrush (48.0%)*
Dunne et al., 2007 [41]	*PGMY09/11 primer sets*	*1,921*	*Women (United States)*	*20 low-risk 23 high-risk*	*Self-collected using a vaginal swab (26.8%)*

Carestiato et al., 2006 [33]	*Hybrid Capture (HC2)*	*5,833*	*5,833 women (Southeastern, RJ, Brazil)*	*5 low-risk 13 high-risk*	*Smears collected using a cervical cytobrush (44.9%)*
Sotlar et al., 2004 [42]	*MY09/11 + GP5+/6+ + NMPCR*	*1,525*	*Women (Germany)*	*05 low-risk 14 high-risk*	*Cervical scrapes (34.7%)*

** indicates the distribution of epidemiological data collected from women across the five Brazilian regions: 48.5% of the data were obtained from the North, Northeast, and Midwest regions, while 51.5% were from the South and Southeast regions.*

*** indicates Spanish acronym for "EStudio multice´ntrico de TAMizaje y triaje del ca´ncer cervicouterino con pruebas de virus PApiloma humano", a study currently being conducted in Argentina, Colombia, Paraguay, Bolivia, Costa Rica, Honduras, Mexico, Peru, and Uruguay* [52].
